# *crystIT*: complexity and configurational entropy of crystal structures via information theory

**DOI:** 10.1107/S1600576720016386

**Published:** 2021-02-01

**Authors:** Clemens Kaußler, Gregor Kieslich

**Affiliations:** a Department of Chemistry, Technical University of Munich, Lichtenbergstrasse 4, 85748 Garching, Germany

**Keywords:** information theory, crystal structure complexity, open source computer programs

## Abstract

The information content of a crystal structure as conceived by information theory has recently proved an intriguing approach to calculate the complexity of a crystal structure within a consistent concept. Given the relatively young nature of the field, theory development is still at the core of ongoing research efforts. This work provides an update to the current theory, enabling the complexity analysis of crystal structures with partial occupancies as frequently found in disordered systems. To encourage wider application and further theory development, the updated formulas are incorporated into *crystIT* (crystal structure and information theory), an open-source Python-based program that allows for calculating various complexity measures of crystal structures based on a standardized *.cif file.

## Abstract 2

Information theory provides an intriguing framework for evaluating the complexity of a crystal structure. This article provides an improvement to the current theory and describes the integration of the updated formulas into an open-source Python-based program called *crystIT*.

## 1. Introduction   

The definition of complexity is a challenging and fascinating subject, touching different scientific disciplines such as economics, informatics, biology, mathematics and chemistry. Instead of defining complexity per se, it is in practice easier to ask ‘Which system is more complex?’, nicely showing that the challenge of defining complexity is closely related to the identification of an appropriate scale to measure complexity. Depending on the scientific area and the type of system, different scales have been proposed, such as dimension, number of unique components or simply human observation, amongst many others. All of these scales come with their own hurdles, such as lack of measurement techniques, definition of unique components and subjectivity, leaving us with the realization that every measuring system for complexity is only useful for a certain observer, in a defined context, for a defined purpose. In this article, the Shannon entropy is used as a measuring system as defined by information theory (Shannon, 1948 ▸), providing us with a framework to differentiate between the complexity of crystal structures as initially introduced by Krivovichev (2014 ▸).

Over the years, the term ‘complex’ has been used various times in the literature for describing crystal structures (Pauling, 1929 ▸; Valenzano *et al.*, 2011 ▸; Loa *et al.*, 2012 ▸), and indeed, parameters such as crystal class, number of different polyhedrons, space group, and number of atoms in the asymmetric unit or in the reduced unit cell are temptingly simple measures. Arguably, a combination of these indices is required to give a full grasp of the depth of crystal structure complexity, but it raises a follow-up question of appropriate weighting factors when one is interested in a quantitative measure. In turn, and not surprisingly, Burdett *et al.* (1994 ▸) came to the conclusion that ‘Complexity is largely a qualitative, frequently intuitive, notion.’ Nevertheless, there was and still is interest in finding (and arguably a need to find) a quantitative concept to evaluate crystal structure complexity. For instance, Baur *et al.* (1983 ▸) defined topological and crystallographic parsimony indices of crystal structures, and more recently, the number of atoms per reduced unit cell was used as a measure to classify various metallic alloys (Dshemuchadse & Steurer, 2015 ▸). Such practical concepts seem suitable for assessing structure complexity within certain material subclasses, but exhibit drawbacks related to a limited discriminating character between simple crystal structures and when one is interested in comparable measures across different material classes. Other approaches are more abstract, such as the algorithmic complexity descriptions based on work by Chaitin (1975 ▸) that was adapted for crystallography by Mackay (2001 ▸) and Estevez-Rams & González-Férez (2009 ▸). For a more detailed overview of attempts to quantify the complexity of crystal structures, we refer the reader to the review by Krivovichev (2014 ▸).

Krivovichev (2014 ▸) applied the concept of Shannon entropy to crystalline materials, evaluating the information content of a crystal structure. Subsequently, he proposed a concise concept that has the potential to capture the full multifaceted challenges of defining the complexity of a crystal structure on the basis of the information content. Recently, Hornfeck (2020 ▸) has suggested a few improvements of the concept, emphasizing the importance of theory development and the current state of research in this relatively young area. Importantly, comparisons between Shannon entropy, crystal structure complexity and configurational entropy can be drawn, opening intriguing opportunities for the assessment of the configurational entropy of crystal structures and its change during phase transitions (Krivovichev, 2016 ▸). In the long term, the concept shows great promise to contribute to a general understanding of crystalline matter and properties, where the quantification of the configurational entropy of a crystal structure shows the greatest potential to close the gap to applied inorganic chemistry.

In this work we follow on from the work of Krivovichev, proposing an updated formula that allows for evaluating the information content, and in turn the complexity, of crystal structures with partially occupied sites and defects. The proposed formula and recent improvements by Hornfeck are incorporated into *crystIT* (crystal structure and information theory), an open source Python-based program. *crystIT* facilitates the application of the approach by non-specialists, the screening of crystallographic databases and method development in general. An intuitive understanding between crystal structure, information content and complexity is then fostered by applying *crystIT* to selected research examples.

## 2. Theory   

Shannon (1948 ▸) introduced a concept to determine the information content of a message, known today as Shannon entropy. Motivated by exploring limits in signal processing, data compression and cryptography, the concept has developed into one of the central pillars in information theory. In the following sections, the framework of Shannon’s entropy is introduced, its application to crystal structures as given by Krivovichev is described, and our and Hornfeck’s improvements to the concept are presented.

### 2.1. Idea and basics of information theory   

Following Shannon, the information *I* contained in each symbol *c* of a message occurring with a probability of *p*_*c*_ is defined by
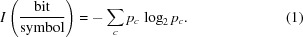
Looking at a standard example, the message ‘Hello’ comprises four distinct letters, occurring with probabilities of *p*_H,e,o_ = 1/5 and *p*_l_ = 2/5. The message’s Shannon information is therefore *I* = 1.9 bit symbol^−1^ and its total information content is calculated by scaling the symbol-wise information content by the number of symbols, *i.e.* *I*_total_ = *I* × 5.

### 2.2. Information theory and crystal structures   

By drawing an analogy between a message consisting of symbols *c* and the reduced unit cell of a crystal structure composed of crystallographic orbits *k*, Krivovichev applied Shannon’s formula to calculate the information content as provided by crystallographic data. Following this train of thought, the probabilities *p*_*c*_ are given by the quotient of the crystallographic orbits’ multiplicities *m*_*k*_ and the number of atoms in the reduced unit cell *v*,
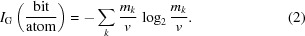
Subsequently, Krivovichev established a correlation between the information content of a crystal structure and its perceived complexity, qualifying *I*_G_ as a quantitative and easily conceivable measure of crystal structure complexity derived from information theory.

Inspecting equation (2) and looking for practical limitations, the question arises as to by which means partial occupancies can be considered. In its current form equation (2) is only suitable for calculating *I*_G_ of crystal structures in which each crystallographic orbit is fully occupied by one atomic species. In other words, the information content as calculated by equation (2) represents the information provided by the decoration of the space group with atomic positions, whereas more information is contained in the specific atoms that fill these abstract positions. Materials that adopt partially occupied positions are common, such as solid solutions with disordered sites as can be found in alloys and various minerals, or for high-temperature disordered phases. The specifics of these partial occupancies become important when looking at the relation between the Shannon entropy and thermodynamic entropy, potentially providing insight into the phase-transition thermodynamics.

#### 2.2.1. Adaptation of the information theory approach to partially occupied sites   

How does one include partial occupancies in formula (2)? Coming back to the linguistic example introduced in Section 2.1, a repeated string of hellos is considered, in which some words are randomly replaced by their German translation: ‘HelloHalloHelloHelloHallo’. According to equation (1) and based on the letters’ occurrence probabilities (*p*_H,o_ = 1/5, *p*_l_ = 2/5, *p*_a_ = 2/25, *p*_e_ = 3/25), the information content per character has increased slightly: *I* = 2.1 bit symbol^−1^.

The smallest repeating unit in this string is equal in size to the string itself, and in turn the string is, strictly speaking, equal in length to its unit cell, which therefore consists of five times as many positions as ‘Hello’. Now, when assuming that this string is repeated with an average probability of ‘Hello’ and ‘Hallo’ equal to 3:2 but with a non-periodic distribution, the scenario is better described by 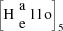
As a bulk analysis technique to obtain crystallographic information, diffraction relies on periodicity, returning averages of positions of disordered atoms (analogous to letters). In other words, we attempt to analyse a five-letter unit cell of the repeating pattern ‘H llo’ that is disordered at the second position with probabilities (or occupancies) *p*_a_ = 2/25 and *p*_e_ = 3/25. Application of equation (2) to this disordered unit cell would erroneously result in the same information content as ‘Hello’, because the calculation is not based on the types of atoms (or characters) or occupancies but only on their positions. Hence, neither solely the atom types (the same element may be involved in entirely different coordination environments) nor the isolated crystallographic orbits (these can be filled partially or by different species) are sufficient for a crystal structure description. Note that this shows striking parallels to the topological index defined by Baur *et al.* (1983 ▸) but augmented by the implementation of information theory and a finer consideration of topology in the form of crystallographic orbits.

Therefore, we propose equation (3), wherein the sum is formed over distinguishable species *t* rather than crystallographic orbits *k*. A species *t* is defined by a unique combination of chemical element or vacancy and crystallographic orbit. The probabilities *p* still reflect the chance of encountering a species *t* when observing a randomly chosen position in the reduced unit cell. However, to consider fractional occupancies, *p* is calculated by the product of the occupied crystallographic orbit’s multiplicity *m* with the respective species’ fractional occupancy value occ, divided by the total number of positions in the reduced unit cell, *P*:
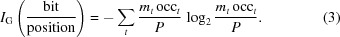
By analogy with the Kröger–Vink notation (Kröger & Vink, 1956 ▸), fractional vacancies are also considered as individual species *t* in equation (3), forming distinct vacancy species for every crystallographic orbit that is only partially occupied by atoms. We will expand on this idea in Section 2.5, but for now we want to highlight that, by including vacancies, the sum over all probabilities is one, as all crystallographic orbits are formally fully occupied and *P* = 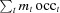 = 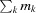. For fully occupied orbits, the number of positions per reduced unit cell *P* is equal to the number of atoms *v*, transforming formula (3) to Krivovichev’s equation [equation (2)]. Notably, aliovalently substituted systems are also included in this approach, since there is no difference in structural information content whether the residual space (1 − occ) of a crystallographic orbit is empty or occupied by a different chemical element.

The information content of the whole reduced unit cell is then calculated by multiplication of *I*_G_ by the total number of positions in the reduced unit cell (red. u.c.),
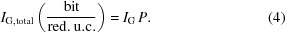
For clarity, we consider the lead zirconate titanate ceramic PbZr_0.35_Ti_0.65_O_3_ as an example. The information relevant to this calculation is provided in Table 1 ▸ (Mir *et al.*, 2007 ▸). A total of *P* = 10 positions are occupied, distributed among three crystallographic orbits, since titanium and zirconium ions occupy a shared 2*a* Wyckoff position. *I*_G_ is calculated by plugging the given probabilities *p* into equation (3), resulting in *I*_G_ = 1.56 bit position^−1^. Following equation (4), *I*_G,total_ = 15.6 bit red. u.c.^−1^ is obtained.

Note that atoms of the same element can be crystallographically nonequivalent. For instance, in yttrium barium copper oxide, YBa_2_Cu_3_O_7−*x*_, there are four crystallographically distinguishable oxygen species (Williams *et al.*, 1988 ▸) and in turn their summands in equation (3) are calculated separately. Additionally, the oxygen position at Wyckoff position 1*e* is only partially occupied (occ = 1 − *x*), so that another term is added for the partial vacancy (occ = *x*). For *x* = 9% the information content consequently amounts to *I*_G_ = 2.96 bit position^−1^.

### 2.3. Extension by Hornfeck   

Recently, Hornfeck (2020 ▸) pointed out that there are various unrelated structures that share the same *I*_G_ as defined by Krivovichev. Yet many of these crystal structures are characterized by different numbers of spatial degrees of freedom of their crystallographic orbits, or ‘site arities’, which are denoted *A* in the context of this paper (*a*_*k*_ for a single crystallographic orbit’s arity, *A* = 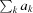). In other words, different materials were observed to have the same information content, although the occupied Wyckoff positions and so the occupied crystallographic orbits had different constraints in their *x*, *y*, *z* coordinates. Therefore, in addition to *I*_G_, Hornfeck proposed the arity-based coordinational complexity, *I*_coor_, in which the sum is formed over individual crystallographic orbits *k*, 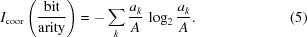
This measure of information essentially contains the information on coordinates that must be defined for the complete description of a crystallographic orbit when its Wyckoff position is known. In order to maintain consistency, Hornfeck subsequently renamed Krivovichev’s information content *I*_G_ as ‘combinatorial’ complexity, 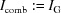, and defined configurational complexity *I*_conf_ as the strong additive sum of coordinational and combinatorial complexities [see Hornfeck (2020 ▸) for the mathematical background]. Using our updated measure for combinatorial complexity (*i.e.* *I*_comb_) and combining it with *I*_coor_, we obtain
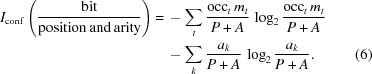
The use of arities as additional information content leads to a more discriminating character of the complexity measure, following a chemist’s intuition. We will pick up on this point when discussing redundancies in Section 2.6. For now we highlight that, in the rest of this work, we will continue to use Krivovichev’s measure *I*_G_. To avoid any ambiguities, both measures are implemented in *crystIT*.

### 2.4. Configurational entropy   

Many different areas in chemistry are united in the quest to understand macroscopic behaviour as a function of microscopic interactions, that is, the identification of structure–composition–property relations. When one is interested in fundamental principles that underlie the formation of (crystalline) condensed matter (Harper *et al.*, 2019 ▸), or temperature- and pressure-dependent properties (Frenkel, 1999 ▸), the entropy *S* is an important parameter that ties the macroscopic to the microscopic world.

Statistically, *S* can be accessed by the Boltzmann formula, 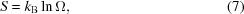
translating the challenge to Ω which ‘counts the ways of finding the internal coordinates of a system for thermodynamically equivalent macroscopic states’ (Fultz, 2010 ▸). *k*_B_ is the Boltzmann constant. A typical simplification to approach Ω, and in turn *S*, in crystalline matter is to divide *S* into several contributions such as *S* = *S*_conf_ + *S*_vib_, with *S*_conf_ the configurational entropy and *S*_vib_ the vibrational entropy.^1^ This simplification is based on the idea that *S*_conf_ is related to the spatial arrangement of atoms which is temperature independent, whilst *S*_vib_ is related to a temperature-dependent contribution describing the movement of atoms which is itself tied to the atomic interactions. Here we only consider the spatial arrangement of atoms and hence the concept of Shannon entropy relates to *S*_conf_. In other words, it seems that information theory can provide us with a concept to calculate *S*_conf_ for crystal structures.

Krivovichev (2016 ▸) followed up on this idea, showing that starting from equation (7) a formula can be obtained in which the information content as provided by crystallographic data contributes negatively to the configurational entropy of the structure: 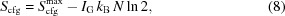
with *S*_cfg_ the configurational entropy, 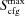 the maximum configurational entropy obtained when all atoms (positions) are symmetrically equivalent, *I*_G_ as obtained from equation (2), *N* the number of atoms in the crystal and ln(2) a conversion factor between binary and natural logarithms, *i.e.* bit and nat. This formula follows the chemist’s intuition that information and entropy are reciprocally related. However, there are some discrepancies in the values that are derived by equation (8), making this area an exciting field of active research. One important aspect is related to the scaling of *S*_cfg_ to formula units rather than atomic sites. Likewise, the entropy of mixing and its increase along a substitution series should be considered, suggesting that *S*_cfg_ itself consists of several contributions.

**Figure 1:**
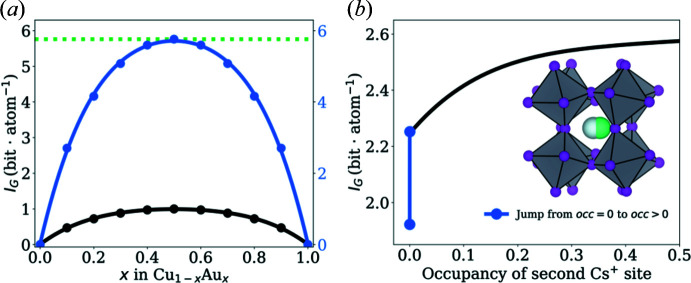
Calculated Shannon entropies *I*_G_ of two examples which were calculated using equation (3) as implemented in *crystIT*. (*a*) The Shannon entropy of the binary solid solution Cu_1−*x*_Au_*x*_. The green line indicates the expected change of *S*_cfg_ as calculated by Boltzmann for a 50:50 alloy. The blue curve can be obtained when multiplying *I*_G_ by *R*ln(2). Notably, the same results are obtained when applying the formula for the entropy of mixing (Gibbs), *i.e.* *S*_mix_ = 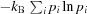 with *p*_*i*_ = *x*_*i*_. (*b*) The Shannon entropy of CsPbI_3_ shows a jump when going from the ordered to the disordered phase, highlighted by the blue line. The inset shows the perovskite structure of CsPbI_3_ with Cs^+^ disordered in the void of the ReO_3_-type network.

Here, we only point out that the calculation of *I*_G_ for the substitution series Cu_1−*x*_Au_*x*_, with 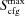 = 0 for the pure elements Cu and Au, is in agreement with the entropy of mixing for a binary alloy after multiplication of *I*_G_ by *R* ln(2) [Fig. 1 ▸(*a*)]. This result motivates further work in this direction and confirms how equation (3) attributes partially occupied sites. For development purposes, the calculation of *S*_cfg_ is implemented in *crystIT* (see Appendix *B* [App appb] for details).

### 2.5. Vacancy   

As stated in Section 2.2.1, vacancies are considered as individual species for information content calculation according to equation (3). This is by analogy with the Kröger–Vink notation, in which vacant sites are denoted 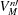 or 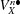 (Kröger & Vink, 1956 ▸). Just as white spaces such as ‘ ’ are necessary for the complete description of a language and contribute to its information content, vacancies *V* are required for the description of defective or dis­ordered structures. Although this approach is entirely logical from an information theory point of view and yields mostly coherent results, the incorporation of vacancies into the calculation has some counterintuitive consequences.

As an example, we discuss the temperature-dependent *I*_G_ values of the perovskite CsPbI_3_. Whilst there is no evidence for disorder at temperatures below 150 K, it was recently reported that above ∼150 K the dodecahedrally coordinatated Cs^+^ cation becomes disordered over two sites [Fig. 1 ▸(*b*)] (Straus *et al.*, 2020 ▸). Intuitively, it would be expected that the information content would rise continuously with increasing occupation of the second site, but a jump in *I*_G_ is observed at the temperature at which the first Cs^+^ occupies the second site (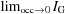). This jump originates from the newly added crystallographic orbit which is immediately filled by a vacancy (1 − occ) [Fig. 1 ▸(*b*)]. Thereafter, the information content behaves as expected. Note that the addition of a new crystallographic orbit draws a clear line to the previously discussed case of Cu_1−*x*_Au_*x*_, where the crystallographic orbit that becomes partially occupied by Cu and Au already exists in the end members. But what is the meaning of this jump in *I*_G_? From information theory the jump in *I*_G_ is expected, since the addition of a new crystallographic orbit contains a considerable amount of information. In fact, it is this piece of information that is key to the crystallographic description of the disordered structure, even for very small occupancies. In turn, the jump in *I*_G_ seems to be in agreement with both information theory and chemistry, acknowledging the additional crystallographic orbit that is required to describe the high-temperature phase of CsPbI_3_.

### 2.6. Redundancy   

Shannon entropy is highest upon equal distribution of atomic species among crystallographic orbits or positions (uniform distribution of probabilities *p*), which can be verified by considering the partial derivatives 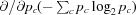 with boundary conditions of 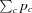 = 1 and *p*_*c*_ ∈ (0, 1]. In turn, the maximum Shannon entropy per character, *I*_G, max_, is given by the logarithm of the message’s total number of unique characters *c*, translating to the number of unique atom species *t* in the reduced unit cell, which is *T*:
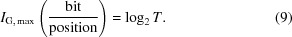
The redundancy *R* is then defined as
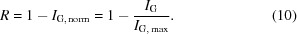



Upon further investigation into the entropy of the printed English language, Shannon (1951 ▸) noted that there are different levels at which the entropy of a language can be estimated. Under the assumption of no knowledge about the language and an analysis of its composition based entirely on strings of meaningless letters, essentially as conducted in this work, the redundancy of English is about 50% because of phenomena or ‘constraints’ such as the necessity of the letter ‘q’ to be followed by ‘u’, a high tendency of ‘h’ to follow ‘t’ or the overall frequent appearance of the letter ‘e’. Although interesting predictions can be derived from rules found by a purely stochastic approach, an even higher level of redundancy of about 75% is estimated when considering grammatical rules and long-range statistical effects in written English. Knowledge of the language therefore enables even better prediction abilities, as demonstrated by Shannon in experiments with native speakers who were supposed to guess missing letters of fill-in-the-blank texts.

By analogy with the application of information theory to crystal structures, Mackay (2001 ▸) wrote that ‘Pauling’s rules reflect chemical experience corresponding to a native knowledge of English in Shannon’s example.’ For instance, an (inorganic) chemist can qualitatively construct the crystal structure of β-crystobalite based on chemical intuition and the information that it adopts a variation of the diamond structure. Whilst such considerations seem to be of a purely scientific nature in the current state, a large redundancy, particularly when combined with the ‘chemist’s grammar’, maybe as represented by Pauling’s rules, might offer new avenues in crystal structure prediction, the identification of ‘wrong’ crystal structures and the subsequent refinement.

Closely related to the topic of redundancy is the question of whether all sources of information have yet been included in equation (3). As mentioned above, Hornfeck provided a recent update to the theory through the incorporation of arities, clearly improving on the discriminating character of different complexity measures. For instance, most of the allotropes of carbon and phosphorous show different *I*_conf_ values, whereas they are largely indistinguishable in *I*_G_.

Looking for potential sources of information that are not yet included, we emphasize that the analysis presented here relies fully on the crystallographic information file (CIF) and therewith on the quality of the structure solution. Moreover, the CIF is an idealized representation of a real crystal structure that exhibits naturally occurring point defects. Acknowledging the constant efforts from computational scientists in obtaining energies for the formation of point defects, opportunities exist to incorporate these in equation (3) via statistical approaches in the future. For instance, when knowing the energy that is necessary to create a Schottky defect in NaCl, it is possible to calculate the defect concentration (partial occupancies) as a function of temperature and in turn the temperature-dependent complexity. Given the increasing notion across various material classes that defects are not independent (Keen & Goodwin, 2015 ▸), it remains an open question as to whether such an extension contains significant meaning. Looking at real crystals, the existence of limited crystal volume, *i.e.* the surface as defect, is an important point and fully neglected in this approach. Although it only seems to be important for particle sizes in the nano-regime, this raises interesting questions in the context of the complexity measure of clusters that consist of a defined number of atoms, *e.g.* the series of neutral and charged gold clusters. Likewise, it is currently unclear how the information content and *I*_G_ develop when transitioning from isolated molecules to molecular crystals or even co-crystallization products, suggesting a role for configurational entropy in crystallization theory.

This brings us to the last important point where our chemical intuition raises a question about the meaning of chemical bonds within information theory, assuming that their existence alters the structure’s information content. On the basis of the information given within a CIF, which is sufficient to recreate a crystal structure, we came to the conclusion that chemical bonds in crystalline solids are redundant information, as these are unequivocally defined by the type of atomic species involved and their positions. Thus, a CIF can be seen as insensitive towards chemistry such as chemical bonds and material class, and so is information theory.

We stress here that the field is still in its infancy, with theory development and questions regarding interpretation limits in the current focus, and the relationship between the configurational entropy of a crystal structure and information theory as given in equation (8) representing a strong motivation.

## 3. The *crystIT* program   

*crystIT* is an open source Python-based program for calculating the information content of crystal structures. The source code is provided as a ready-to-use Python file, is freely available (see Appendix *B* [App appb] and the GitHub repository https://github.com/GKieslich/crystIT for further details) and is based on the formulas as given in Section 2.

**Figure 2:**
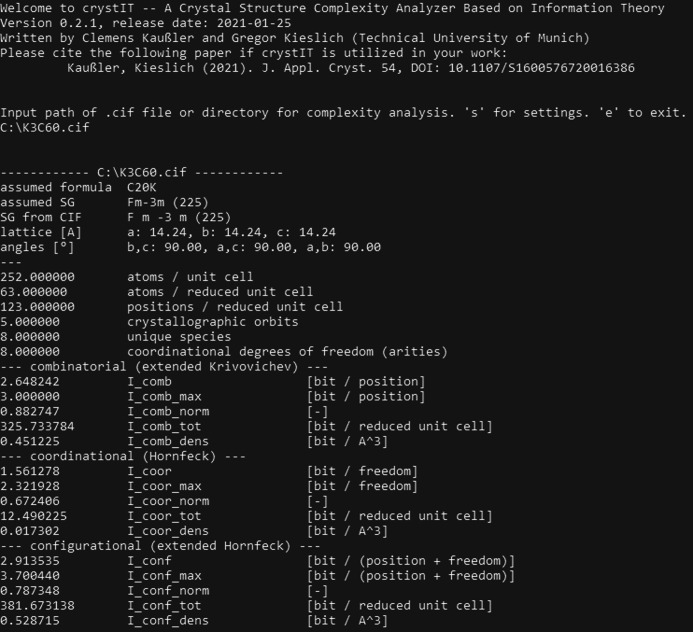
An example output of *crystIT*, as run in single-file mode for K_3_C_60_. In batch-file mode, a *.csv file is generated containing the output data.

As input, *crystIT* requires a standardized CIF. In single-CIF mode the program returns the calculated parameters directly into bash; see Fig. 2 ▸ for the output for K_3_C_60_ (Stephens *et al.*, 1991 ▸). In batch mode a CIF-containing directory is passed to the program and the script outputs a *.csv file containing the different complexity measures. The batch mode is set up for large data set processing and supports multi-threading for better performance. The menu provides access to on-the-fly occupancy editing and options to alter settings regarding symmetry tolerance, recursive sub-directory scanning, the number of threads in batch mode, switching between comma and dot as decimal separator, and the output of entropy parameters derived from equation (8).

In attempts to identify potential problems with the program, we observed erroneous space-group detection in some cases, which can be circumvented by altering the symmetry tolerance value. We also came across CIF parsing errors in rare cases, which can be fixed by re-exporting the file from *VESTA* (Momma & Izumi, 2011 ▸). For better identification of such cases, error messages are given as output in bash or the *.csv files.

## 4. Results and discussion   

Having described the mathematical foundation of *crystIT*, we now proceed to investigate chemical interpretations of *I*_G_. By looking at the complexity of the crystal structures for some selected examples, this section aims to create a more intuitive picture between information theory and crystal structure complexity.

### 4.1. Screening of the Crystallography Open Database   

Krivovichev (2014 ▸) performed a database analysis based on crystallographic data as available in the Inorganic Crystal Structure Database (http://icsd.fiz-karlsruhe.de/icsd/). He correlated *I*_G_ with *I*_G, total_, compared different measures of complexity and evaluated complexity for various inorganic material classes. In order to provide a different research angle, and to show the big-data analysis capabilities of *crystIT*, we here focus on the development of complexity with time, using the full Crystallography Open Database (COD; http://www.crystallography.net/cod/; Gražulis *et al.*, 2009 ▸) as input. The COD is an ‘open-access collection of crystal structures of organic, inorganic, metal–organics compounds and minerals, excluding biopolymers’ and the complete data set consists of approximately 440 000 CIFs (60 GB) as of June 2020. The data set was batch-processed in about six hours using *crystIT* on a single workstation, demonstrating the scalability and robustness of the program.

**Figure 3:**
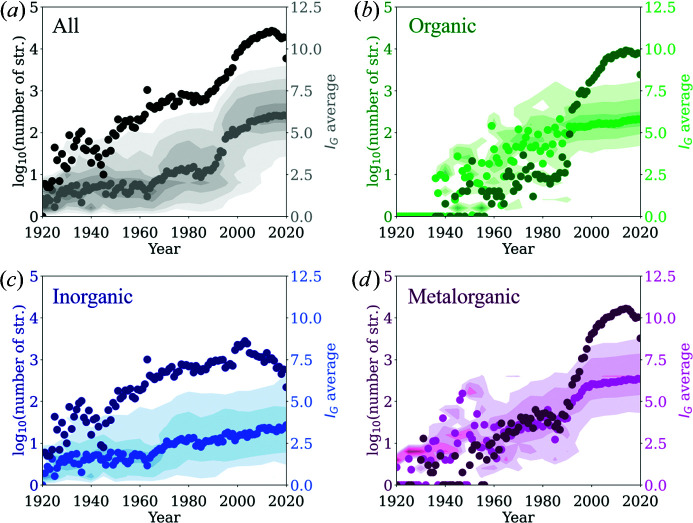
Information content screening of the COD data set (June 2020), showing the number of structures and average information content for (*a*) all structures in the database and the subsets of (*b*) organic, (*c*) inorganic and (*d*) metal–organic structures. The contour plots in the background represent the frequency of how the information content is distributed, showing ∼85% (or more) of the underlying data set. Note that, for the subsets of organic and metal–organic structures, these frequencies are only representative starting from 1987 and 1963, respectively.

Database screening studies rely heavily on the quality and number of data entries. Therefore, an initial assessment of the number of published structures as a function of year is important [Fig. 3 ▸(*a*)]. The overall exponential increase in available crystal structures is testimony to the growing number (and efficiency) of research capabilities, which affects the field of crystallography as an indispensable analysis tool for synthetic chemistry in various areas. The sharp decrease in the number of structures per year between 2014 and 2020 reflects the delay between the publication of crystal structures and their incorporation into the database. Given that the number of structures is still reasonably large, it can be assumed that the database entries are sufficient for qualitative trend evaluation of crystal structure complexities. For interpretation purposes, we have divided the initial data set into three subsets: (i) 55 867 structures without carbon atoms, (ii) 132 165 structures which contain exclusively C, H, N, P, O, S, Se, F, Cl, Br and I, and (iii) the remaining 232 577 structures. These subsets reflect the commonly accepted categorization of materials into (i) in­organic, (ii) organic and (iii) metal–organic materials. Interestingly, this categorization already reveals that the large increase in database entries in the 1990s [Fig. 3 ▸(*a*)] is caused mainly by organic and metal–organic structures, as for in­organic structures only a linear increase is observed between the 1940s and 2000.

In the next step, the development of the annually averaged *I*_G_ for the different subsets is assessed [Figs. 3 ▸(*b*)–3 ▸(*d*)]. The subsets of organic and metal–organic structures behave differently compared with the subset of inorganic structures. For both subsets the averaged *I*_G_ shows a linear increase with a current average *I*_G_ of ∼6–6.5 bit atom^−1^. This trend only appeared around the 1990s, which is presumably related to the significant increase in the number of organic and metal–organic structures deposited in the database since the 1990s. This increase is difficult to attribute to a single factor, but the development of computer technologies, the rise of synchrotrons as highly brilliant light sources for X-ray diffraction, the availability of neutron sources, and advances in detector and laboratory X-ray technologies are all important aspects that have allowed more efficient access to structures with light elements and larger unit cells. For the inorganic subset, a small but linear increase in average *I*_G_ is observed since 1920, and the average and maximum *I*_G_ are smaller compared with the other two subsets. The contour plots of *I*_G_ distribution versus time (background plots in Fig. 3 ▸) show that the discovery of less complex crystal structures, *i.e.* structures with *I*_G_ < 4 bit atom^−1^ for organic and metal–organic and *I*_G_ < 2 bit atom^−1^ for inorganic, has decreased significantly compared with the reporting of structures with larger information content. For instance, in all years after 1990 over 85% (or more) of the structures deposited in the organic subset show *I*_G_ values larger than 4.5. Furthermore, structures with *I*_G_ > 9 bit atom^−1^ are still uncommon. It will be interesting to see how this develops further over time.

The most complex structures found in this screening have complexities of around *I*_G_ ≃ 11.5 bit atom^−1^ and were discovered within the past five years. Many of these structures seem highly complex, such as supramolecular arrays of helical oligoamides which self-assemble around a linear rod-like oligocarbamate (Wang *et al.*, 2017 ▸), and various coordination cages and multimetallic complexes with large *I*_G_ values. Additionally, there are examples that have been assigned a large *I*_G_ due to their large unit cells, in which assemblies of smaller subunits such as an eightfold polycatenated hydrogen-bonded and π-stacked framework of 1,3,5-tris(4-carboxyphenyl)benzene (Zentner *et al.*, 2015 ▸) can be observed. Such examples that are clearly composed of sub-units seem to show an intrinsically large compressibility when considered in relation to the spatial orientation and sequence of such subunits to each other. However, any clear symmetric relation between these subunits is captured within the crystal structure file and in turn in the results of information theory. In any case, it seems that, for such materials, using *I*_G_ as the complexity measure can lead to counterintuitive results – counterintuitive when compared with chemical intuition. The algorithmic complexity approach put forward by Chaitin (1975 ▸) would improve on this discrepancy between calculated and perceived complexities, but a new complexity descriptor for molecules (how should one define a ‘subunit’?) and a measure for the three-dimensional molecular alignment in the reduced unit cell would need to be generated.

An interesting example in this context that reveals a certain subjectivity of chemical intuition as a measure of complexity is proteins, which show *I*_G_ values larger than any structures discussed herein. Depending on the focus, proteins can be described through only a few letters, and if needed, additional details on the structural arrangement can be provided in various levels of depth. The approach applied in this work focuses strictly on the information content provided by the CIF, and in the presence of many symmetrically independent atoms and large unit cells, as in the case of proteins, large *I*_G_ values are obtained. This example demonstrates that the concept discussed here should be seen as a concise language which comes with its own subjectivity, determined by the amount and type of information which the calculation is based on. Depending on the research example, this might or might not be in agreement with chemical intuition.

### 4.2. Silicon carbide polytypes   

**Figure 4:**
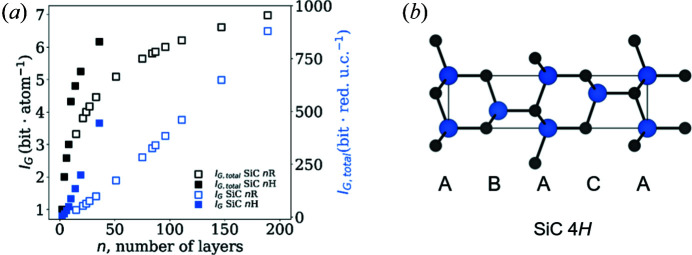
Analysing the complexity of various silicon carbides as a function of number of layers. (*a*) Complexity is plotted as a function of layers of various silicon carbide polytypes. (*b*) The structure of SiC 4*H* as viewed along the *b* axis, with labelled *ABAC* layering. Si atoms are in blue and C in black. Depending on the number of layers *n* in a given silicon carbide, a six- or threefold axis is present, which is reflected in the complexity measure and shows the close relation between complexity and symmetry.

In a footnote, Pauling (1929 ▸) mentioned that, by varying the order of close-packed *ABC* layers, infinitely many combinations ‘with ever increasing complexity’ are possible. Inspired by this note and motivated to test *I*_G_ against Pauling’s statement, we chose silicon carbides as our next example. Many different polytypes of silicon carbide are known, which differ only in the order in which C_1/2_–Si–C_1/2_ slabs rotate around a *C*_3_ axis, giving rise to (hypothetically) infinitely large unit cells (Parthé *et al.*, 1993 ▸). For instance, SiC 2*H* has an *AB* order, SiC 4*H* *ABAC*, SiC 6*H* *ABCACB* and so on [*cf.* Fig. 4 ▸(*b*)].

The information content and calculated complexity do indeed rise with the number of layers [Fig. 4 ▸(*a*); *I*_G_ approximately logarithmic and *I*_G, total_ slightly faster than linear]. However, it is also clear that the rise in information content of rhombohedral polytypes occurs at a lower rate than for those that can be described by a hexagonal lattice (note that rhombohedral lattices are typically observed when the number of layers is a multiple of three). Looking for the origin of this phenomenon, it can be observed that in the rhombohedral cell there are two additional lattice points compared with a hexagonal Bravais lattice. In turn, only two-thirds of the crystallographic orbits required for the description of SiC in the hexagonal case are necessary when describing SiC rhombohedrally. Upon closer inspection, a kink in the *I*_G_ development of SiC *nH* is visible between eight and ten layers. This is also related to different relative numbers of crystallographic orbits that must be defined depending on the space group (hexagonal 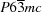, one per layer; trigonal *P*3*m*1, two per layer).

Although the general trend is in agreement with the intuitive understanding of crystal structure complexity, the differences related to the rhombohedral and hexagonal series are at minimum counterintuitive. In the context of information theory, however, this result is expected and, at the current state of research, seems to be an intrinsic artefact when using crystallographic orbits as a measure of complexity calculations of crystal structures.

### 4.3. Ruddlesden–Popper series   

The series of Ruddlesden–Popper (RP) oxides is another interesting example and conceptually related to silicon carbides through the idea of increasing complexity via the incorporation of layers with varying repetition units. The structure of RP oxides is built from 2D slabs of perovskite unit cells with unit-cell thicknesses *n*. These slabs are sandwiched between rock salt (*AX*) layers to form RP oxides with the general formula *A*_*n*+1_ *B*_*n*_ *X*_3*n*+1_. Importantly, for 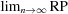 the perovskite structure *ABX*_3_ is obtained.

Similar to the silicon carbide example, an infinite number of crystal structures can in principle be envisioned based on the variation of *n*. Intuitively we therefore expect an increase in complexity with increasing *n*, although experimentally known RP examples do not exceed *n* = 3. We chose Sr_*n*+1_Ti_*n*_O_3*n*+1_ and Rb_*n*+1_Cd_*n*_Cl_3*n*+1_ and calculated the series’ complexities (Table 2 ▸). All RP phases in Table 2 ▸, including the prototypical perovskite when treated as *n* = 0, demonstrate a direct proportionality between complexity and number of layers: *I*_G_ ∝ *n* (*R* ^2^ = 0.998). In contrast to the *ABX*_3_ compounds with fluorides, which typically adopt the perovskite structure, RbCdCl_3_ crystallizes in a structure containing double rutile-like columns of CdCl_6_ that are linked by Rb atoms (Natarajan *et al.*, 1978 ▸). Therefore, a different complexity compared with the rest of the RP phases is obtained for RbCdCl_3_, showing that *I*_G_ depends on factors beyond the empirical formula.

Surprisingly, other related compounds that do not satisfy the general RP formula but crystallize in similar structures, such as the oxyhalide Ca_2_CuCl_2_O_2_ or distorted variations, *e.g.* La_2_CuO_4_, show complexities equal to those of the canonical RP phases. Even the complexities of anion-deficient *M*_2_CuO_3_ with *M* = Ca^2+^ or Sr^2+^ do not differ much.

The RP series is therefore a beautiful example in which the intuitive understanding of complexity is well matched by the complexity values calculated from information theory.

### 4.4. Perovskite tilt systems   

Taking a closer look at the iconic material class of perov­skites, it is interesting to look for correlations between tilt systems and complexity as represented by *I*_G_. For the classification of perovskite tilts after the Glazer (1972 ▸) notation we refer the reader to some insightful book chapters and reviews (Shimakawa, 2017 ▸; Woodward, 1997 ▸).

A selection of tilt phases for NaNbO_3_ as a phase-rich example are given in Table 3 ▸. Intuitively, we would assign the highest complexity to the phase with three tilts of different magnitudes. Our intuition is challenged when considering the *a* ^−^ *a* ^−^ *a* ^−^ tilt system. Although representing three activated tilts, the tilts are of the same magnitude and direction (as required through symmetry). In turn, one can argue that *a* ^−^ *a* ^−^ *a* ^−^ and *a* ^0^ *a* ^0^ *a* ^0^ are of similar complexity, given that the numbers of different tilt angles are equal. Complexities obtained by information theory confirm this perspective (Table 3 ▸). Furthermore, it seems that the trend as expected from intuition holds for other examples such as KMnF_3_ and CaTiO_3_.

Therefore, the perovskite phases highlight the subtle differences between symmetry and complexity, a difference that was not so clear from the example of silicon carbides. However, this is a far from exhaustive study and it will be interesting to see how complexities of perovskites develop when considering examples with Jahn–Teller active *B*-site cations or other structural distortions, although this is beyond the scope of the present study.

## 5. Concluding remarks   

In conclusion, we have introduced an update to the Krivovichev measure of crystal structure complexity to crystal structures with partial occupancies. For better applicability by non-specialists and for theory development in the future, we have incorporated the concept into *crystIT*, a Python-based program that allows for calculating the complexity of crystal structures on the basis of CIFs.

Looking at the discussed examples, we can observe a few counterintuitive consequences of the utilization of crystallographic orbits for complexity calculations. For instance, we can find a pronounced space-group dependency as observed for silicon carbides, and discontinuous behaviour of *I*_G_. Evidently, further progress is necessary in this direction, either to elucidate these phenomena or to provide further adjustments to the calculations. The general tenor is therefore that theory development is at the heart of ongoing research activities. It is important to remember that the outcomes are only as reliable and accurate as the source of information, in this case the reliability of the crystallographic data as provided through the CIF.

In attempts to identify the potential of the approach, a breakthrough in the calculation of configurational entropy based on crystallographic data clearly has the potential to bring the concept of Shannon entropy closer to applied materials science. Potential research directions might be a more quantitative analysis of calorimetric data to extend our understanding of phase-transition thermodynamics in in­organic materials and coordination polymers alike. Likewise, we have mentioned the calculation of complexities of clusters based on information theory, but why stop at periodic matter? The elucidation of quasicrystals’ complexities seems a difficult but scientifically intriguing future task.

## Figures and Tables

**Table 1:** Crystallographic information for PbZr_0.35_Ti_0.65_O_3_ at 300 K, space group *R*3*c*

Species number *t*	Element	Wyckoff position	Occupancy (occ)	*p* = *m*_*t*_ occ_*t*_/*P*
1	Pb	2*a*	1.00	0.20
2	Ti	2*a*	0.65	0.13
3	Zr	2*a*	0.35	0.07
4	O	6*b*	1.00	0.60

**Table 2:** Complexity calculations for some selected RP phases with a focus on series based on SrTiO_3_ and RbCdCl_3_ *I*_G_ values shown in italics were not used in the regression which is mentioned in the text, since RbCdCl_3_ does not crystallize in a perovskite structure and the anion-deficient compounds are only close to the RP information content.

*n*	RP	Related compounds	*I*_G_ (bit atom^−1^)
0	SrTiO_3_^*a*^		1.37
0	RbCdCl_3_^b^		*2.32*
1	Sr_2_TiO_4_^*c*^	Ca_2_CuCl_2_O_2_*^h^	1.95
1	Rb_2_CdCl_4_^*d*^	La_2_CuO_4_^+*i*^	1.95
1		Ca_2_CuO_3_^#*j*^, Sr_2_CuO_3_^#*k*^	*1.92*
2	Sr_3_Ti_2_O_7_^*e*^	Ba_3_In_2_Cl_2_O_5_*^l^	2.42
2	Rb_3_Cd_2_Cl_7_^*f*^		2.42
3	Sr_4_Ti_3_O_10_^*g*^		2.91

Variations from canonical RP series: * oxychloride, + distorted, # anion-deficient. CIFs sourced from (*a*) Al-Shakarchi & Mahmood (2011 ▸), (*b*) Natarajan *et al.* (1978 ▸), (*c*) Miwa *et al.* (2007 ▸), (*d*) Kruglik *et al.* (1989 ▸), (*e*) Lukaszewicz (1959 ▸), (*f*) Villars & Cenzual (2012*a* ▸), (*g*) Villars & Cenzual (2012*b* ▸), (*h*) Grande & Müller-Buschbaum (1977 ▸), (*i*) Grande *et al.* (1977 ▸), (*j*) Teske & Müller-Buschbaum (1971 ▸), (*k*) Teske & Müller-Buschbaum (1969 ▸), (*l*) Gutau & Müller-Buschbaum (1990 ▸).

**Table 3:** Calculated complexities for some perovskites and their underlying tilt systems

Compound	Space group	Tilt	*I*_G_ (bit atom^−1^)
NaNbO_3_^*a*^	221, 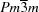	*a* ^0^ *a* ^0^ *a* ^0^	1.37
NaNbO_3_^*b*^	161, *R*3*c*	*a* ^−^ *a* ^−^ *a* ^−^	1.37
NaNbO_3_^*c*^	127, *P*4/*mbm*	*a* ^0^ *a* ^0^ *c* ^+^	1.92
KMnF_3_^*d*^	140, *I*4/*mbm*	*a* ^0^ *a* ^0^ *c* ^−^	1.92
CaTiO_3_^*e*^	62, *Pnma*	*a* ^+^ *b* ^−^ *b* ^−^	1.92
NaNbO_3_^*f*^	63, *Cmcm*	*a* ^0^ *b* ^−^ *c* ^+^	2.52

Crystallographic information was obtained from (*a*) Barth (1925 ▸), (*b*) Seidel & Hoffmann (1976 ▸), (*c*) Darlington & Knight (1999 ▸), (*d*) Asbrink & Waskowska (1994 ▸), (*e*) Buttner & Maslen (1992 ▸), (*f*) Darlington & Knight (1999 ▸).

## Footnotes

1 There are other potential sources of entropy such as charge carriers, magnetic moments and nuclear spins that we neglect at this point.

## Appendix A Utilized software and databases

The crystal structures for the calculations were obtained from either the Crystallography Open Database (COD) (Gražulis *et al.*, 2009 ▸, 2012 ▸, 2015 ▸; Merkys *et al.*, 2016 ▸) or the Cambridge Structural Database (CSD) (Groom *et al.*, 2016 ▸) in the form of CIFs. Some CIFs had to be generated from the original publications. The CIF generation process and the creation of crystal structure images were performed in the *VESTA* software suite (Momma & Izumi, 2011 ▸).

The provided Python (Van Rossum & Drake, 2009 ▸) program requires the *Atomic Simulation Environment* (*ASE*) library (Larsen *et al.*, 2017 ▸) for CIF parsing, and *Spglib* (Togo & Tanaka, 2018 ▸), *PyXtal* (Fredericks *et al.*, 2019 ▸) and *NumPy* (Walt *et al.*, 2011 ▸) for symmetry calculations.

## Appendix B Quick-start guide to the Python program

The open-source program *crystIT* can be downloaded free of charge from https://github.com/GKieslich/crystIT together with an extensive readme file. *crystIT* is written in Python 3 and is therefore compatible across multiple platforms. Package dependencies are described in Appendix *A* [App appa] and in the readme file.

As input, *crystIT* requires a valid path to either a CIF or a directory containing CIFs (batch mode). Depending on the input, it either outputs the information parameter directly to bash (single file) or creates a character-separated value file (.csv) in the directory (batch mode).

The settings can be accessed by typing ‘s’ and confirming with Enter. By activating the recursive subdirectory scan (‘r’), subfolders are scanned in batch mode. The maximum number of threads for multiprocessing in batch mode is automatically set to the maximum number of available threads, but can be adjusted by integer input. The occupancy options (‘o’) allow for on-the-fly occupancy editing in single-file processing. A float input changes *symprec* which defines the tolerance in Cartesian coordinates for *Spglib* to find symmetry: |*x*′ − *x*| < *symprec*. Entropy calculation is activated with ‘s’ and the decimal separator can be toggled between dot and comma by typing ‘d’. Finally, the menu is exited with ‘e’.
